# High Level of Multidrug-Resistant Gram-Negative Pathogens Causing Burn Wound Infections in Hospitalized Children in Dar es Salaam, Tanzania

**DOI:** 10.1155/2021/6644185

**Published:** 2021-07-02

**Authors:** Fatima Kabanangi, Agricola Joachim, Emmanuel James Nkuwi, Joel Manyahi, Sabrina Moyo, Mtebe Majigo

**Affiliations:** ^1^Department of Microbiology and Immunology, Muhimbili University of Health and Allied Sciences, Dar es Salaam, Tanzania; ^2^Department of Microbiology and Immunology, College of Health and Allied Sciences, University of Dodoma, Dodoma, Tanzania

## Abstract

**Background:**

Bacterial infection remains the most common cause of morbidity and mortality in pediatric patients with burn wounds. The increase in infection and multidrug-resistant (MDR) pathogens necessitates a periodic review of antimicrobial susceptibility patterns in the burn units. The study aimed to determine the magnitude of multidrug-resistant Gram-negative (MDRGN) bacteria in children with burn wound infections and describe the resistance patterns in the tertiary and regional hospitals in Dar es Salaam, Tanzania.

**Materials and Methods:**

The study was a hospital-based cross-sectional study design conducted between May 2017 and February 2018. Bacterial isolates from 103 wound swabs of pediatric patients with burn wounds were identified using conventional methods and API 20E. The antimicrobial susceptibility pattern was determined by the Kirby–Bauer disc diffusion method. Data were analyzed using Statistical Package for Social Science (SPSS) version 23.0.

**Results:**

A total of 136 pathogenic Gram-negative organisms were isolated from burn wound infections in pediatric patients. The most isolated Gram-negative bacterium was *Pseudomonas aeruginosa* (39.0%), followed by *Acinetobacter* spp. (28.7%) and *Klebsiella* spp. (16.2%). MDRGN strains made up 80.1% of all Gram-negative isolates. All (100%) *Klebsiella* spp. and *E. coli* were MDR, while 69.2% and 79.2% of *Acinetobacter* spp. and *P. aeruginosa*, respectively, displayed MDR strains. We observed high levels of resistance to commonly prescribed antibiotics. Among *P. aeruginosa* isolates, highest resistance (81.8%) was seen toward meropenem and piperacillin, 79.5% of *Acinetobacter* spp. showed resistance to aztreonam, while 93–100% of *Klebsiella* spp and *E. coli* displayed resistance to amoxyclavulanic acid, ceftriaxone, and ceftazidime. The proportion of extended-spectrum beta-lactamase producers among *Enterobacteriaceae* was 78.6%. There was a significant higher rate of infection with MDRGN organisms in pediatric patients with a higher percentage of total burn surface area (TBSA) than patients with lower TBSA (*p* = 0.016).

**Conclusions:**

*P. aeruginosa*, *Acinetobacter* spp., and *Klebsiella* spp. are the common Gram-negative pathogens causing burn wound infections in hospitalized pediatric patients in our setting. A high proportion of these organisms were multidrug resistant. The findings appeal for regular antimicrobial resistance surveillance in burn wound infection to inform empirical therapy.

## 1. Introduction

Burn injuries are a global public health problem and still remain the leading cause of disability and unintentional death [1, 2]. According to the World Health Organization (WHO), an estimated 180,000 deaths occur globally every year as a result of burns. The majority of these deaths occur in low and middle-income countries, with two-thirds being in African region. Furthermore, in this region, the incidence of death due to burns in children under five is over twice the incidence in children under five worldwide [1]. In 2009, a community-based study conducted among children and adolescents in Dar es Salaam, Tanzania, found that 16.3% of reported injuries were burns [2].

Burn wound infection (BWI) remains one of the most common causes of morbidity and mortality in burn patients [3]. The risk of infections is relatively high due to the immunosuppressing effect of burns, invasive therapeutical procedures, and length of hospitalization [4]. In pediatric burn patients, mortality rates due to sepsis still remain high. Infected burn wound serves as an important source for most of the cases of sepsis [5]. A study conducted in Iraq among pediatric burn patients reported a significant relationship between wound infection and death in 37.7% of the deaths recorded. The deaths were attributed to inappropriate antibiotic use due to delays in laboratory testing including wound swab and blood culture that might have resulted in septicemia and eventually death [6]. In studies involving pediatric patients, sepsis was found to be the leading cause of death in Mozambique (100%) and the second cause of death in Cameroon (24%) [7, 8].

Virus, fungi, and Gram-positive and Gram-negative bacteria are all known to cause infections in burn wound patients [6, 9]. However, Gram-negative organisms have become significant agents of infections in vulnerable burn patients due to their multidrug resistance nature which possess critical therapeutic challenges [10]. Several studies conducted around the world have identified *Pseudomonas aeruginosa*, *Escherichia coli, Acinetobacter baumannii, Klebsiella pneumoniae*, and *Proteus mirabilis* as the most common multidrug-resistant Gram-negative bacteria (MDRGNB) in BWI [4, 10–17]. Most cases of sepsis in burn patients occur as a result of infected burn wounds where *P. aeruginosa* has been found to be the most common organism [5, 18]. Moreover, infections caused by *Pseudomonas* + *Klebsiella* and *Acinetobacter* + *Klebsiella* have been recognized as the major cause of increased mortality and morbidity among pediatric patients [19, 20]. Additionally, burn wound colonization with MDR *Enterobacteriaceae* has been associated with high mortality in hospitalized children [21]. However, it is important to note that the etiology and profile of resistance can vary from one healthcare setting to another [13, 16].

The development of antibiotic resistance causes a big challenge in the treatment of bacterial infections in both adult and pediatric patients. Furthermore, resistance to multiple antibiotic classes reduces the probability of adequate empirical coverage, with possible unfavorable outcomes [22]. Vulnerability to infections and increasing antibiotic resistance among organisms put burn patients at high risk of infection by multidrug-resistant (MDR) organisms [23]. Hospital cleaning practices, antibiotic therapy without knowledge of circulating bacterial strains, and excessive and prolonged use of antibiotics have led to the development and selection of multidrug-resistant bacteria [24, 25]. The MDRGNB has become increasingly common in hospital settings, necessitating the understanding of institutional specific circulating strains [13, 15, 22, 26, 27]. Moreover, healthcare professionals managing burn patients require in-depth knowledge of bacteria causing infection and their antimicrobial resistance patterns to direct empirical therapy [28]. Information on the extent of infections caused by MDRGNB in burn patients is scarce in Tanzania. With this in mind, we undertook the current study to determine the MDRGNB, causing wound infections in hospitalized pediatric burn patients and describe the antimicrobial resistance patterns.

## 2. Materials and Methods

### 2.1. Study Design, Settings, and Population

The study was a hospital-based cross-sectional design conducted at one tertiary hospital, Muhimbili National Hospital (MNH), and two regional referral hospitals (Mwananyamala and Temeke) in Dar es Salaam, Tanzania. The study recruited hospitalized children with burn injury between May 2017 and February 2018. Eligible clients had a clinical diagnosis of burn wound infection based on criteria stated by Appelgren et al., 2002, and the Center for Disease Control (CDC) [29, 30]. Only patients whose parents/guardians gave written informed consent were enrolled.

### 2.2. Sample Size and Sampling Procedure

The sample size for the current study was estimated using the formula for sample size calculation for cross-sectional studies [31], considering 83.3% prevalence of burn infection in Ethiopia [32] and a 7% margin of error. The study employed convenient sampling to recruit eligible clients consecutively until it reached a representative sample size of 103 participants. All participants were examined to establish the presence of eligibility criteria of the burn wound infection, including-presence pus, foul-smelling discharge, blister, change in burn wound appearance or character, increased bleeding tendency, or signs of inflammation.

### 2.3. Sample Collection and Transportation

Before specimen collection, the wound was cleaned with normal saline solution. Two wound swab specimens were then aseptically collected from the depth of the wound using a sterile cotton swab by rotating with sufficient pressure. The specimens were placed in Stuart's transport media and transported to the testing laboratory at Muhimbili University of Health and Allied Sciences (MUHAS) within eight hours of collection.

### 2.4. Bacteria Identification

The Gram staining was performed on one swab to check for the quality of the specimen, presence of bacteria, and pus cells. The other swab was inoculated on MacConkey agar and blood agar (Oxoid, UK) and incubated in ambient air at 35–37°C for 18–24 hours. Preliminary identification of bacterial isolates was made based on colonial morphology, pigmentation, and changes in physical appearance in differential media and Gram stain reaction. The isolates were further identified by using a series of biochemical tests such as Kligler iron agar, sulphur indole motility, Simon's citrate agar, and urease as well as catalase for *Acinetobacter* spp. and the oxidase test for *Pseudomanas* as previously described [20, 33]. Additionally, API 20E (BioMerieux, France) was used on *Enterobacteriaceae*, whose identity could not be obtained by the conventional biochemical tests [19].

### 2.5. Antimicrobial Susceptibility Testing

Antimicrobial susceptibility testing was performed using the Kirby–Bauer disk diffusion method, with commonly prescribed antibiotics, according to Clinical and Laboratory Standards Institute (CLSI) guidelines [34]. At least one antibiotic from the CLSI recommended classes was tested. Briefly, colonial suspension from pure culture comparable to the 0.5 McFarland standard was inoculated on Mueller–Hinton agar. The plates were then incubated at 37°C for 18–24 hours, and the zone of inhibition was interpreted according to CLSI guidelines [34]. *P. aeruginosa* ATCC 35218 and *E. coli* ATCC 25922 were used as the quality control organism. The following antibiotic disks (Oxoid, UK) were tested: ciprofloxacin (5 *μ*g), gentamicin (10 *μ*g), sulphamethoxazole-trimethoprim (1.25/23.75 *μ*g), ceftriaxone (30 *μ*g), ceftazidime (30 *μ*g), imipenem (10 *μ*g), aztreonam (30 *μ*g), piperacillin (100 *μ*g), and meropenem (10 *μ*g).

Extended-spectrum beta-lactamase (ESBL) production was screened by the disk diffusion method on Mueller–Hinton agar using ceftazidime (30 *μ*g) or ceftriaxone (30 *μ*g). Isolates with zones of inhibition <22 mm for ceftazidime and ≤25 mm for ceftriaxone were confirmed by the modified double disk synergy test [35]. Briefly, a lawn culture of test organism was made on a Mueller–Hinton agar plate. Amoxicillin-clavulanate (20/10 *μ*g) was placed in the center of the plate. Ceftazidime (30 *μ*g) and cefotaxime (30 *μ*g) disks were placed 20 mm apart, center-to-center to that of the amoxicillin-clavulanate disk. A distortion or increase in the zone of inhibition towards the disk of amoxicillin-clavulanate was a confirmation of positive ESBL production. *Klebsiella pneumoniae* ATCC 700603 acted as a control strain for a positive ESBL production.

Multidrug-resistant (MDR) was defined as resistance to at least one agent in three or more antimicrobial classes [36]. Antimicrobial classes tested included cephalosporin (ceftazidime, ceftriaxone); aminoglycosides (gentamicin); fluoroquinolones (ciprofloxacin); folate pathway inhibitors (sulphamethoxazole-trimethoprim); carbapenems (imipenem, meropenem), penicillin (piperacillin), and monobactam (aztreonam).

### 2.6. Data Analysis

Statistical analysis was performed using SPSS version 23 (Armonk, N.Y: IBM Corp). Descriptive analysis for categorical variables was summarized in form or frequencies and percentages. Chi-square or Fisher's exact test was used to examine the group comparison. The level of statistical significance was set at *p* < 0.05.

### 2.7. Ethical Approval

Ethical clearance to undertake this study was obtained from the Senate Research and Publications Committee, the Institutional Review Board of MUHAS. Permission to conduct the study was sought from the authorities of MNH, Mwananyamala Regional Hospital, and Temeke Regional Hospital. Study participants' legal guardians provided written informed consent before enrollment.

## 3. Results

### 3.1. Characteristics of Pediatric Patients Enrolled in the Study

A total of 103 pediatric patients with a clinical diagnosis of burn wound infections were enrolled in the study. The median age of the patients was two years, with a range of one month to 10 years. The ratio of males to females was almost equal, and the majority 88 (85.4%) of the patients were from the MNH pediatric burn unit. Antibiotic use was recorded in 60 (58.3%) patients, and 68 (66%) were hospitalized for one week or less before recruitment to the study (Table 1).

### 3.2. Pattern of Bacteria Causing BWI

**Figure 1:**
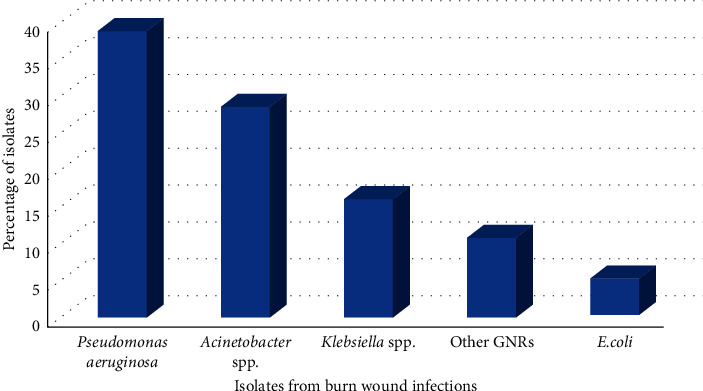
Distribution of aerobic Gram-negative pathogenic bacterial isolates from children with burn wound infections. GNR, Gram-negative rod. Other GNRs are *Enterobacter* spp., *Proteus* spp., *Rahnella aquatilis*, *Raoultella* spp., and *Stenotrophomonas* spp.

Of the 103 pus samples collected, 96/103 (93.2%) had significant bacterial growth. Of these, 61/96 (63.5%) showed multimicrobial infection, while the monomicrobial infection was seen in only in 35/96 (36.5%) samples. In total, 185 bacteria isolates were obtained. Of these, 136 (73.5%) were aerobic Gram-negative bacteria. The rest (26.5%) were Gram-positive bacteria. The most commonly isolated Gram-negative rods were *P. aeruginosa* 53/136 (39.0%), followed by *Acinetobacter* spp. 39/136 (28.7%) and *Klebsiella* spp. 22/136 (16.2%). Other organisms were isolated, although in low frequencies (Figure 1).

### 3.3. Antibiotic Resistance Pattern

The antibiotic resistance profiles of the isolates to commonly used antibiotics are given in Table 2. Generally, isolates showed high resistance to most of the antibiotics tested. The resistance of *P. aeroginosa* ranged from 69.8% for ceftazidime to 81.8% for meropenem and piperacillin. *Acinetobacter* spp. also exhibited high resistance to aztreonam (79.5%), sulphamethoxazole-trimethoprim (77.8%), and to third-generation cephalosporins (66.7% and 67.6%), but resistance to meropenem (18.1%) and imipenem (23.1%) was low. The resistance of *Klebsiella* spp. to third-generation cephalosporins ranged between 93% and 96%. All *E.coli* isolates were resistant to third-generation cephalosporins and sulphamethoxazole-trimethoprim. The proportion of other GNR isolates resistant to the tested antibiotics was also very high. ESBL-producing *Enterobacteriaceae* made up 78.6% (33/42) of all *Enterobacteriaceae* isolates.

### 3.4. Multidrug-Resistant Gram-Negative Bacteria

Of all Gram-negative bacterial isolates from children with BWI, 109/136 (80.1%) were MDR strains. All *Klebsiella* spp. and *E. coli* were MDR, while 69.2% and 79.2% of *Acinetobacter* spp. and *P. aeruginosa*, respectively, displayed MDR (Table 3).

### 3.5. Pediatric Patient Characteristics and MDRGNB

A total of 64 (62.7%) children had burn wound infection with MDRGNB. The proportion of children with infection by MDRGNB was not significantly different between the two age groups of less or equal to five (63%) and above five years (54.5%), *p* > 0.5. There was no difference in the proportion of infection with MDRGNB between males (61.5%) and females (62.7%) (*p* > 0.5). Thirty-six (60%) of the children with a history of antibiotic use before specimen collection had infection with MDRGNB, which did not differ from those without prior antibiotic use (*p* = 0.597). Concerning admitting hospitals, both MNH and Temeke had a higher proportion (65.9%–66.7%) of MDRGNB infection compared to Mwananyamala (22.2%) (*p* = 0.035). There was a significant higher rate of infection with MDRGN organisms in pediatric patients with a higher percentage of total burn surface area (TBSA) than patients with lower TBSA (*p* = 0.016). Those with extended hospital stay show more infection with MDRGN organisms; however, the difference was not statistically significant (*p* > 0.05) (Table 4).

## 4. Discussion

Multidrug-resistant Gram-negative bacteria are increasingly becoming problematic in many burn units across the globe. In the present study, we evaluated the pattern of Gram-negative pathogens from children with burn wound infections and their antibiotic resistance patterns. The most commonly isolated Gram-negative bacteria were *P. aeruginosa*, followed by *Acinetobacter* spp. Similar findings of the predominance of *P. aeruginosa* have been reported in other studies [12, 14, 37]. *Acinetobacter* spp. has emerged in the current study as an essential cause of hospital-acquired infections in burn patients. The finding is in contrast with similar studies in other countries that reported low frequencies of this bacterium [38, 39]. A study in Nigeria on the Gram-negative pathogens, causing burn wound infections, reported *Klebsiella* spp. as the predominant pathogen [40]. In this study, other Gram-negative rods such as *E. coli*, *Proteus mirabilis*, and *Enterobacter* spp. were also observed in very low frequencies, similar to reports from other studies [8, 10]. The differences in the causative bacteria of BWI could be explained by the difference in geographic environment and infection control measures [12].

We observed high levels of resistance (53.3–100%) among *Enterobacteriaceae* isolates towards commonly prescribed antibiotics which including amoxicillin-clavulanate, ciprofloxacin, gentamicin, imipenem, sulphamethoxazole-trimethoprim, and to all third-generation cephalosporins. The observed trend is similar to the previous reports from Tanzania [41–43] though from different clinical conditions. High resistance (59.1–66.7%) towards ciprofloxacin observed in the current study is slightly higher than previously reported (20–56%) on similar isolates from the same settings [42]. Resistance to imipenem was also high (62.5–100%) among *Enterobacteriaceae* isolates and *P. aeruginosa,* as found by a study on MDRGNB in Mwanza, Tanzania [44]. The high resistance could signify an increase in the spread of carbapenemases-producing pathogens in our hospital settings. Carbapenem is considered the last line antibiotic to treat patients with MDR bacterial infections. The high levels of resistance to carbapenem bring challenges to the management of patients with limited options that are more toxic and less effective. The considerable implications occur in low to middle-income countries like Tanzania as it is a threat to public health and requires active detection and infection control measures.

Antibiotic resistance was markedly high (54.5–81.7%) among the nonfermentative Gram-negative bacteria, namely, *P. aeruginosa* and *Acinetobacter* spp. *Acinetobacter* spp. were relatively resistant to all antibiotics tested. Nevertheless, they showed low resistance to imipenem (23.1%) and meropenem (18.1%). These findings are in agreement with previous reports in our settings [42]. Similarly*, P. aeruginosa* isolates had high resistance (69.8–81.8%) to the following: third-generation cephalosporins, piperacillin, ciprofloxacin, and aztreonam. Of note is the relatively high resistance to the carbapenems, findings that concur with a study from other countries [27]. The resistance could indicate an increase in carbapenemase-producing *Pseudomonas*. The previous study in the same settings reported imipenem as the antibiotic of choice for the treatment of *P. aeruginosa* infections [42]. The contrast signifies a change in resistance patterns of *P*. *aeruginosa.*

*Enterobacteriaceae* registered high resistance (53.3–100%) with third-generation cephalosporins, which can be explained by the presence of extended-spectrum beta-lactamase (ESBL) enzymes in most (78.6%) of the isolates. A similar finding was reported in our settings [45, 46]. Studies have shown that bacteria encode ESBL genes on plasmids, which also carry other resistance genes [47], and this may explain the presence of resistance to multiple antibiotics among ESBL isolates in this study. The overall proportion of MDRGNB isolates in this study was very high (80.1%). The trend of increased rates of MDRGNB isolates from burn wounds is similar to a study performed in Ethiopia [48]. Notably, all *Klebsiella spp* and *E. coli* isolates were MDR.

No proportion difference of infection with MDRGNB was observed among children enrolled in this study in relation to age and sex, a finding similar to a study in Palestine [49]. Similarly, a nonsignificant difference was observed among children with or without prior antibiotic use. In contrast, another study in Tanzania reported an increased proportion of infection with MDR bacteria among participants with prior antibiotic use [42]. We observed a higher proportion of infection by MDRGNB in children with more extensive burns and a trend of more infection with MDRGNB in children with extended hospital stays. We hypothesize that children with extensive burns stay longer in the hospital, which puts them at an increased risk of infection by MDR bacteria, as reported in the USA [23] and Colombia [50]. A significant difference in infection with MDRGNB in different admitting hospitals could be explained by the vast disparity in hospital environment and practice of infection prevention and control.

The limitation of this study is that we did not perform molecular techniques to confirm the phenotype of MDR amongst the isolates. Genotyping of the circulating organisms is very important as it would allow us to know the mechanism of resistance and their mode of spread. In turn, it has significant consequences in the development of detection and control strategies.

## 5. Conclusion

This study found a high level of multidrug-resistant Gram-negative bacteria in hospitalized pediatric patients with burn wound infection. The common MDRGN bacteria causing burn wound infections in these children included *P. aeruginosa*, *Acinetobacter* spp., and *Klebsiella* spp. Regular surveillance, in vitro antimicrobial testing and monitoring is necessary to guide empirical therapy in pediatric burn patients. The practices would in turn curb the emergence of multidrug-resistant organisms and decrease morbidity and mortality attributed to these infections.

## Figures and Tables

**Table 1:** Characteristics of pediatric patients with burn wound infections.

Variables	Number	Percentages (%)
Age group (yr)		
≤5	92	89.3
>5	11	10.7

Gender		
Male	52	50.5
Female	51	49.5

Hospital		
MNH Pediatric Burn Unit	88	85.4
Temeke Pediatric Ward	6	5.8
Mwananyamala Pediatric Ward	9	9.0

Antibiotic use before specimen collection		
Yes	60	58.3
No	43	41.7

The extent of burn (% TBSA)^*∗*^		
≤10%	35	34.0
11–20%	30	29.1
≥21%	19	18.4

Length of hospitalization before recruitment		
≤1 week	68	66.0
2 weeks	18	17.5
≥3 weeks	17	16.5

^*∗*^The extent of the burn was not recorded in 19 participants and hence omitted. TBSA, total burn surface area.

**Table 2:** Antimicrobial resistance pattern of Gram-negative bacteria isolated from children with BWI.

Bacteria isolates (*n*)	Antimicrobial agent resisted (%)									
	AMC	CRO	CAZ	CIP	CN	SXT	IMP	MEM	PRL	ATM
*Acinetobacter* spp. (*n* = 39)	—	67.6	66.7	60	60	77.8	23.1	18.1	54.5	79.5
*P. aeruginosa* (*n* = 53)	—	—	69.8	79.2	79.6	—	79.2	81.8	81.8	79.2
*Klebsiella* spp. (*n* = 22)	95.5	92.9	95.5	59.1	81.8	83.3	62.5	—	—	—
*E. coli* (*n* = 7)	100	100	100	57.1	80	100	66.7	—	—	—
Other GNRs (*n* = 15)	80	70	53.3	66.7	66.7	100	100	—	—	—

AMC, amoxycillin clavulanate; ATM, aztreonam; CAZ, ceftazidime; CIP, ciprofloxacin; CN, gentamicin; CRO, ceftriaxone; IMP, imipenem; MEM, meropenem; PRL, piperacillin; SXT, sulfamethoxazole-trimethoprim.

**Table 3:** Multidrug resistance of Gram-negative bacteria isolated from children with BWI.

Bacteria	Class of antimicrobial resisted, *N* (%)					Total, *N* (%)
	*R*3	*R*4	*R*5	*R*6	*R*7	
*P. aeruginosa*	1 (2.4)	6 (34.0)	16 (38.1)	19 (45.2)	—	42 (79.2)
*Acinetobacter* spp.	5 (18.5)	4 (14.8)	10 (37.0)	6 (22.2)	2 (7.4)	27 (69.2)
*Klebsiella* spp.	8 (36.4)	6 (27.3)	8 (36.4)	—	—	22 (100)
*E. coli*	2 (28.6)	4 (57.1)	1 (14.3)	—	—	7 (100)
Other GNRs	2 (18.2)	3 (27.3)	6 (54.5)	—	—	11 (73.3)

*R*3–*R*7, resistant to 3, 4, 5, 6, or 7 classes of antimicrobials tested.

**Table 4:** Proportion of multidrug resistance and patient characteristics.

Characteristics	Frequency	Infection with MDRGN, *N* (%)	*P* value
Overall	103	64 (62.1)	

Age group (years)			0.583
≤5	92	58 (63.0)	
>5	11	6 (54.5)	

Gender			0.899
Males	52	32 (61.5)	
Females	51	32 (62.7)	

Admitting hospital			0.035
MNH Pediatric Burn Unit	88	58 (65.9)	
Temeke Pediatric Ward	6	4 (66.7)	
Mwananyamala Pediatric Ward	9	2 (22.2)	

Antibiotic use before specimen collection			0.597
Yes	60	36 (60.0)	
No	43	28 (65.1)	

The extent of burn (% TBSA)			0.016
≤10%	35	19 (54.3)	
11–20%	30	26 (86.7)	
≥21%	19	14 (73.7)	

Length of hospitalization before recruitment			0.163
≤1 week	68	39 (57.3)	
2 weeks	18	11 (61.1)	
≥3 weeks	17	14 (82.3)	

## Data Availability

All relevant data generated and analyzed during this study are available from the corresponding author upon request.

## Abbreviations

BWI: Burn wound infection
CLSI: Clinical and Laboratory Standards Institute guidelines
ESBL: Extended-spectrum beta-lactamase
GNR: Gram-negative rods
MDR: Multidrug resistant
MDRGNB: Multidrug-resistant Gram-negative bacteria
MNH: Muhimbili National Hospital
MUHAS: Muhimbili University of Health and Allied Sciences
WHO: World Health Organization.
